# Towards Neoatherosclerosis: A Porcine Model for Enhanced Device Testing

**DOI:** 10.1007/s12265-026-10778-4

**Published:** 2026-05-26

**Authors:** Jing Xie, Ole Gemeinhardt, Stephanie Bettink, Ralf Hauptmann, Mathias Schannor, Melanie Loechel, Ulrich Speck, Tobias Haase

**Affiliations:** 1 Department of Radiology, Charité - Universitätsmedizin Berlin, corporate member of Freie Universität Berlin, Humboldt-Universität zu Berlin, Charité Campus Mitte, Charitéplatz 1, 10117 Berlin, Germany; 2 InnoRa GmbH, Robert-Koch-Platz 4, 10115 Berlin, Germany; 3 Clinical and Experimental Interventional Cardiology, University of Saarland, 66421 Homburg Saar, Germany; 4 Bundesanstalt für Materialforschung und -prüfung (BAM), Richard-Willstätter-Straße 11, 12489 Berlin, Germany

## Abstract

The porcine in-stent stenosis model has been essential for developing drug-coated devices but does not replicate the hyperlipidemic, pro-atherogenic conditions driving neoatherosclerosis. We aimed to establish a practical porcine model for testing endovascular therapies targeting neoatherosclerosis. Juvenile pigs were fed a high-fat, nicotine-supplemented diet (HFDN). After two weeks, bare-metal stents were implanted in coronary and peripheral arteries; controls received a standard diet. Four weeks later, stented, and non-stented artery segments were analyzed. HFDN feeding induced elevated serum LDL and cotinine. At follow-up, in-stent late lumen loss was significantly greater in peripheral arteries (A. iliaca: 2.3 ± 0.6 vs. 1.1 ± 0.5 mm; A. femoralis: 3.4 ± 1.2 vs. 2.0 ± 0.4 mm), but not coronary arteries. HFDN-fed pigs showed distinct signs of early neoatherosclerosis, including peri-strut foam cell accumulations and atheroma formation. Elemental imaging and chemical analysis confirmed increased calcium phosphate deposition in arteries of HFDN-fed pigs. This straightforward protocol enables testing of devices aimed at preventing or treating neoatherosclerosis.

**Supplementary Information:**

The online version contains supplementary material available at 10.1007/s12265-026-10778-4.

## Introduction

The introduction of drug-eluting stents (DES) has significantly reduced restenosis rates compared to bare-metal stents (BMS). However, the antiproliferative drugs employed to inhibit neointima formation compromise endothelial function, creating inflamed vessel wall segments that are particularly susceptible to neoatherosclerosis (NA). NA is characterized by the accumulation of lipid-laden foam cells within the neointimal tissue of stented arteries, forming a pro-inflammatory environment that promotes the formation of vulnerable plaques [1]. This presents significant challenges for medical devices aimed to treat *de novo* stenosis and in-stent restenosis, highlighting the need for novel intervention strategies [2, 3]. Developing such treatments requires animal models that accurately replicate the conditions promoting NA in patients undergoing endovascular revascularization.

The porcine in-stent stenosis model was foundational in the development of DES and the drug-coated balloon (DCB) [4]. It relies on the proliferation of smooth muscle cells, leading to neointimal thickening as a response to injury from vascular stretch and stent implantation. While it mimics the key processes of restenosis, it fails to replicate the clinical scenario of endovascular interventions in a hyperlipidemic, pro-atherogenic environment. To bridge this translational gap, several modifications have been proposed to better align preclinical studies with patient conditions. These models typically combine vascular injury with diet-induced hyperlipidemia in pigs [5, 6] and in rabbits [7–9], or achieve enhanced hyperlipidemia through genetic silencing of lipid transport proteins [10, 11]. While these models offer advantages for specific research questions, their practicality is constrained by extended induction periods (pig and rabbit), limited vascular treatment sites per animal or restriction to large central vessels (rabbit), substantial costs, and limited commercial availability (genetically modified pigs) [12].

Given the context, our objective was to establish a model in young domestic pigs to study endovascular therapies in an environment prone to NA formation. To achieve this, we combined an atherogenic diet (high in fat, cholesterol, and cholate) with pro-atherogenic factors (nicotine, calcium, phosphate, and methionine) in domestic pigs to rapidly induce an environment conducive to NA following stent implantation. Here, we describe the development and characterization of this high-fat diet plus nicotine (HFDN) model, detailing its effects on blood chemistry, vascular remodeling, and the formation of early NA lesions 4-weeks post-intervention.

## Materials & Methods

### Study Design and Intervention

All animal studies were conducted in accordance with the guidelines of the European Commission Directive 86/609/EEC and the German Animal Protection Act, based upon the Animal Ethics Committee approvals (Saxony–Anhalt, Germany). The in vivo study was performed in 10 juvenile domestic male pigs (*n* = 5 per group; mean body weight 28.3 ± 1.4 kg).

**Fig. 1:**
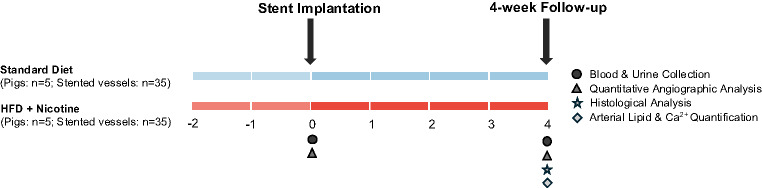
Experimental design of the study. Implantation of a bare metal stent with vessel overstretch was done 2 weeks after diet initiation. At 4-weeks after stent implantation (4-week follow-up) angiography was done, animals were sacrificed, and tissues collected. Lipid and Ca^2+^ quantifications were done in untreated artery segments

Animals in the control group (standard diet) received conventional piglet diet (deuka primo plus, deuka Deutsche Kraftfutterwerke GmbH & Co., Düsseldorf/Könnern, Germany, diet composition provided in Supplemental Table S1). Animals in the experimental group (HFDN) were fed a high-fat diet supplemented with 30 ppm nicotine (C 9020, Altromin, Lage, Germany; diet composition provided in Supplemental Table S1). All animals received 1 kg of the diet per day, corresponding to approximately 1 mg nicotine per kg body weight per day for the experimental group. HFDN feeding started 2 weeks before the initial vascular intervention, which consisted of balloon-expandable stent implantation with approximately 20% vessel overstretch in selected coronary and peripheral arterial segments. Feeding continued until the end of the study at 4 weeks post-intervention. Except for the dietary composition, all animals received identical treatment following standard protocols for anesthesia and interventional procedures (see Fig. 1; detailed in Supplemental Method M1), consistent with our previous published methodology [13].

Bare metal stents were implanted in three coronary arteries (left anterior descending [LAD], left circumflex [LCX], right coronary artery [RCA]; 3.0 or 3.5 × 20 mm, Rebel, Boston Scientific, MA, USA), bilateral internal iliac arteries (5.0 × 19 mm, Express Vascular SD, Boston Scientific, MA, USA), and bilateral femoral arteries (6.0 × 18 mm, euca PW, Eucatech, Weil am Rhein, Germany). Stent deployment pressures were individually adjusted by the operator to achieve a target vessel overstretch of approximately 20%, based on angiographically measured reference vessel diameters and the manufacturers’ balloon compliance charts, utilizing inflation pressures between 8 and 12 bar (detailed in Supplemental Table S2). Angiographic assessment of treated arterial segments was performed pre-, post-implantation and at 4-week follow-up (FU).

### Blood and Urine Analyses

Arterial blood samples were obtained via the arterial sheath before the initial intervention (two weeks after HFDN diet initiation) and at 4-week FU before control angiography. Analyses included blood chemistry (phosphate, calcium, and vitamin D), hematological parameters (complete blood count), serum lipids (HDL, LDL, triglycerides, non-esterified fatty acids), and serum cotinine content using an ELISA Kit (Cotinine ELISA Kit, KA0930, Abnova, Taipei, Taiwan). Urine samples were collected from the bladder at sacrifice for cotinine analysis.

### Quantitative Angiography

Offline quantitative angiography (QA) was performed on angiograms taken before, during, and immediately after treatment, as well as at 4-week FU. Analysis was conducted using QAngio XA software, (Medis, Leiden, the Netherlands), following standardized protocols for vessel diameter measurements and late lumen loss (LLL) calculation.

### Histological Analyses

Treated arteries were dissected and fixed in 10% buffered formalin. Stented arterial segments were dehydrated using a Histokinette processor (Leica TP1020, Nussloch, Germany), infiltrated in methyl-methacrylate and embedded according to the manufacturer’s protocol (Technovit 9100, Heraeus Kulzer, Hanau, Germany). Three segments (proximal, mid, distal) within each stented area were sectioned with a coping saw and re-embedded in methyl-methacrylate. Tissue sections of 8 μm thickness were prepared using a rotary microtome equipped with a tungsten carbide knife (D-profile, Leica, Nussloch, Germany), mounted on microscopy slides (Superfrost Plus, Thermo Fisher, Schwerte, Germany), and deplasticized with methoxyethyl acetate. Sections were stained with Movat pentachrome (Article No. 12057, Morphisto, Offenbach am Main, Germany) according to the manufacturer’s protocol. Stained arterial cross-sections were digitized with a Hamamatsu slide scanner and evaluated with Hamamatsu NDP viewer software (Hamamatsu Photonics, Herrsching, Germany). The following parameters were quantified: lumen area, neointimal area (calculated as internal elastic lamina [IEL] minus lumen area), and percent lumen loss (calculated as [neointimal area/IEL area] x 100). Semi-quantitative injury scores were assessed according to Schwartz et al. [14], and fibrin scores were evaluated using a modified protocol based on Kamann et al. [15]. These scores were determined for each stent strut site, averaged per section and stent. Additionally, semi-quantitative scoring for peri-strut foam cells presence was done as follows: 0 = no foam cells around the strut; 1 = < 50% circumferential foam cells around the strut; 2 = ≥ 50% circumferential foam cells around the strut. Collagen staining was carried out using picro-sirius red (PSR) according to the manufacturer’s protocol (Article No. 13422, Morphisto, Offenbach am Main, Germany). Immunofluorescence staining for macrophages was conducted with mac-2 antibody (clone M3/38 unconjugated, Cedarlane, Burlington, Canada) as previously described [15].

### Laser Ablation Inductively Coupled Plasma Mass Spectrometry (LA-ICP-MS)

Laser ablation inductively coupled plasma mass spectrometry (LA-ICP-MS) measurements were performed on a NWRimage laser ablation system (266 nm, Elemental Scientific Laser, Bozeman, USA) equipped with a low-dispersion ablation cell in a TwoVol3 ablation chamber attached to an ICP-ToF-MS (Model 2R, Tofwerk AG, Thun, Switzerland) using a 1.016 mm ID PEEK tubing and a Dual Concentric Injector 2 (DCI2). Instrumental settings are summarized in Supplemental Table S5. Raw data processing was performed using Iolite v4 software (Elemental Scientific Laser, Bozeman, USA) following established protocols [16, 17]. Complete analytical procedures are described in Schannor et al. 2025 [18].

### Arterial Lipid and Calcium Quantification in Unstented Vessels

Arterial segments from the external and internal iliac arteries (*A. iliaca*), femoral arteries (*A. femoralis)*, internal thoracic arteries *(A. thoracica)*, and abdominal aorta were harvested and stored at -20 °C. Frozen arterial segments were rinsed with distilled water and dried with lint-free paper towels before weighing. Tissues were subsequently freeze-dried using a lyophilizer (Alpha 1–4 LSC, Christ, Osterode am Harz., Germany) for 24 h until constant weight was achieved. Freeze dried arterial segments were re-weighted to determine tissue dry mass and water content. For lipid quantification, dried tissues were incubated twice for 24 h each in 40 mL of methanol-chloroform (1:1 v/v) at room temperature. The combined extracts were dried at 105 °C overnight until constant weight was obtained to calculate the percentage of lipids per tissue dry mass. For calcium analysis, the same arterial segments (following lipid extraction) were treated with 1 mL concentrated hydrochloric acid for 30 min at 80 °C. Extracts were slowly concentrated by heating to the boiling point, and the residual matter was mixed with water and filtered through a 0.2 μm filter. Photometric determination of the calcium content was performed by mixing 10 µL of sample solution with 1 mL arsenazo(III) solution (200 µmol/L arsenazo(III) in 0.1 mol/L 2-(N-Morpholino) ethanesulfonic acid buffer). Following 5 min incubation, absorbance was measured at 650 nm with a 1 cm plastic cuvette. Calcium content was calculated per unit tissue dry weight.

### Statistical Analysis

Body weight development was analyzed by two-way ANOVA with factors “group” and “time”, followed by Sidak’s multiple comparison test. Blood parameters were analyzed using a mixed-effects model with factors “group” and “time”, followed by Sidak’s multiple comparison test. QA derived variables were averaged per treatment group for statistical comparison. Histomorphometric variables of the three cross-sectional planes (proximal, mid, distal) were averaged to obtain a mean value per stented segment, subsequently averaged per arterial bed (coronary arteries, internal iliac arteries, femoral arteries) and compared between treatment groups. Arterial calcium and lipid content values were pooled across all analyzed arteries, averaged per animal, and then compared between treatment groups. Comparisons were performed using two-tailed, unpaired t-tests for normally distributed data or Mann-Whitney tests for non-normally distributed data. Data are presented as mean value ± standard deviation (SD) where applicable. All statistical analyses were conducted with GraphPad Prism (Version 8, GraphPad Software, CA, USA). Statistical significance was defined as *p* ≤ 0.05.

## Results

### Treatment and Observations

**Fig. 2:**
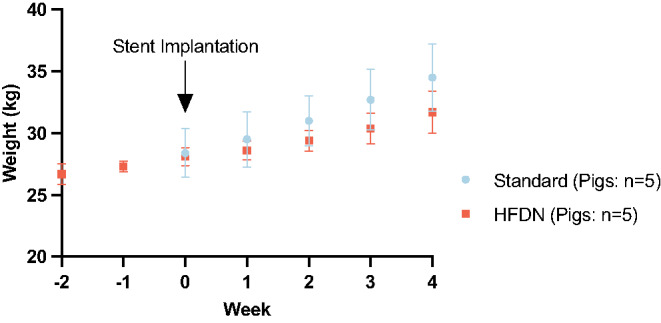
Body weight development during the study. *N* = 5 pigs per group at stent implantation shown as mean ± SD

Pigs received 1 kg/day high-fat diet supplemented with 30 ppm nicotine (HFDN) that corresponds to approximately 1 mg nicotine/kg body weight/day or the same amount of standard diet (Standard). At the time of stent implantation, both groups exhibited similar body weight (Fig. 2, Week 0). Treatment of all 10 pigs was performed according to the established study protocol under general anesthesia, with successful angiographic guidance and bare metal stent implantation with controlled vessel overstretching in both coronary and peripheral arteries. No unexpected adverse reactions were observed. Spasms due to mechanical irritation by the guide wire, stent implantation, and overstretch were observed in some arteries with no difference in frequency between the groups. Post-interventional angiography confirmed vessel patency, adequate stent expansion, and appropriate stent apposition to the vessel wall in all treated vessels. During the following 4 weeks period, standard diet-fed pigs showed faster weight gain compared to the HFDN group without reaching statistical significance until the end of the study (Fig. 2).

One animal in the HFDN group died one day before scheduled re-angiography at the 4-week FU. This animal had shown no prior signs of illness, pain or abnormal behavior throughout the study period. Gross examination revealed no pathological changes. This animal was excluded from blood analyses (4-week FU), angiographic datasets (implantation and 4-week FU) and the histological fibrin scoring in the femoral arteries. At 4-week FU angiography, all arterial segments in the remaining animals were patent. No obvious deterioration of electrocardiographic parameters or heart rate was observed at any time point. Systolic and diastolic blood pressures showed no significant differences between treated groups (HFDN vs. Standard) nor between baseline and 4-week FU measurements (see Supplemental Table S3).

### Hematology and Biochemical Parameters

**Fig. 3:**
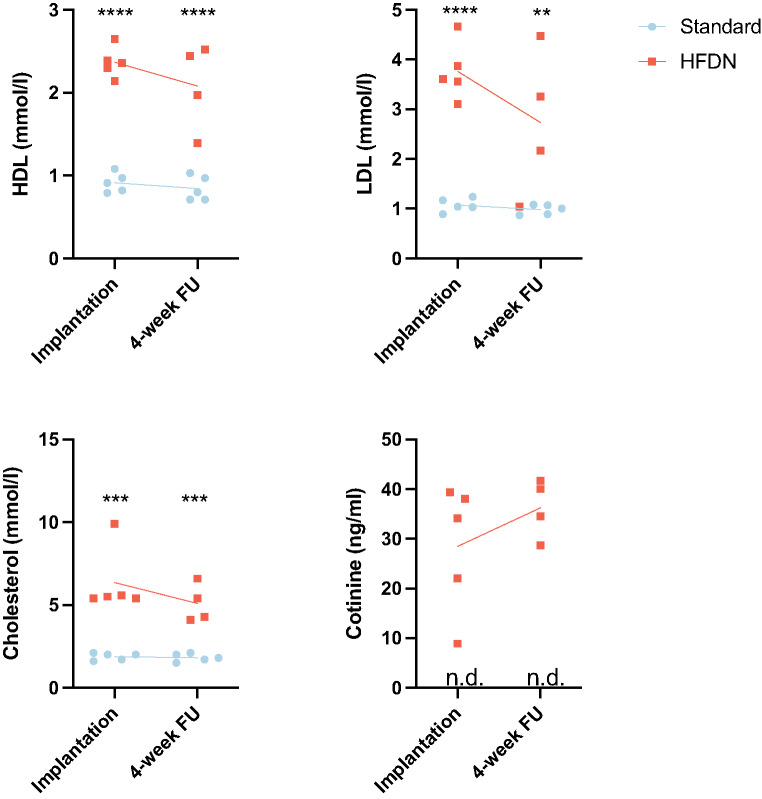
Serum HDL, LDL, cholesterol and cotinine levels at stent implantation and at 4-week follow-up (FU). Mean and individual data points. *N* = 5 pigs per group at stent implantation, at 4-weeks follow-up *n* = 5 pigs in the Standard group and *n* = 4 pigs in the HFDN group. Cotinine was not detected (n.d.) in standard diet fed animals (detection limit 1 ng/ml). ** *p* < 0.01, ***p < 0.001, ****p < 0.0001

At stent implantation (2 weeks after HFDN initiation), HFDN animals showed significantly increased HDL, LDL-, and total cholesterol serum levels compared to the standard group (Fig. 3 and Supplemental Table S4), while hematological parameters were not significantly different between groups. At 4-week FU, HFDN animals maintained significantly elevated HDL, LDL, total cholesterol, and free fatty acids serum levels compared to the standard group (Fig. 3 and Supplemental Table S4). Hematological analysis revealed significantly decreased erythrocyte counts and hemoglobin concentrations in HFDN animals compared to the standard diet group. The number of thrombocytes were significantly increased in HFDN animals compared to the standard group. Serum phosphate and calcium levels were not significantly different between treatment groups and time points. Serum vitamin D levels could not be analyzed in 5 of 10 samples at the stent implantation and are therefore not shown. At 4-weeks FU, serum vitamin D levels did not differ significantly between groups (Supplemental Table S4). Cotinine, the primary metabolite of nicotine, was detected in HFDN group serum samples at both intervention and 4-week FU but was not detected in standard diet group samples. Urine cotinine concentrations were significantly elevated in HFDN animals compared to standard diet-fed pigs (Fig. 3 and Supplemental Table S4), confirming systemic nicotine exposure and metabolism.

### Quantitative Angiography

**Fig. 4:**
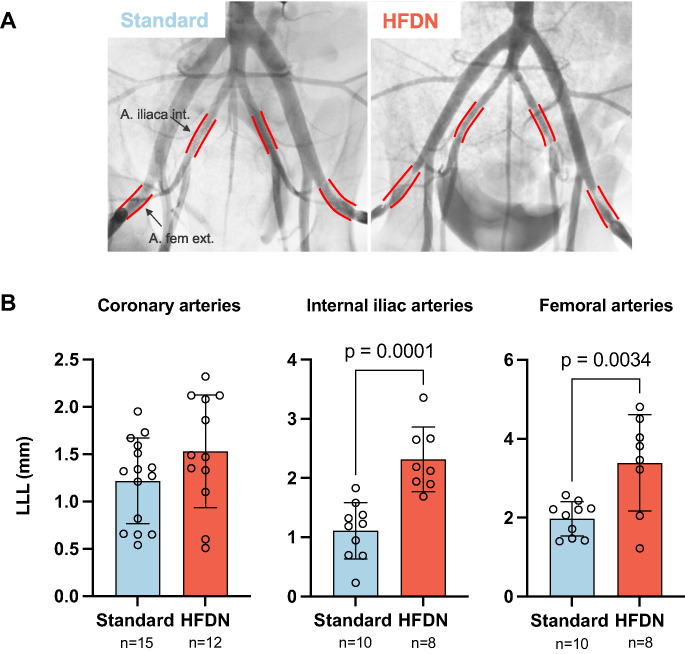
(A) Angiograms of stented segments (red) in peripheral arteries at 4-week FU. (B) In-stent late lumen loss (LLL) at 4-week FU of stented coronary- and peripheral artery segments. Mean ± SD and individual data points. For coronary arteries *n* = 15 stents in the Standard group and *n* = 12 stents in the HFDN group. For peripheral arteries *n* = 10 stents in the Standard group and *n* = 8 stents in the HFDN group

Immediately following stent implantation (post-intervention), in-stent lumen diameters were similar between standard- and HFDN-fed animals across all vascular beds (Table 1). At the 4-week FU, in-stent lumen diameters were consistently smaller in the HFDN groups compared to the standard diet groups. HFDN feeding led to significantly greater LLL in peripheral arteries but not in coronary arteries compared to standard diet fed pigs (Table 1; Fig. 4). Specifically, internal iliac arteries in HFDN animals demonstrated a LLL of 2.32 ± 0.55 mm compared to 1.11 ± 0.47 mm in the standard group (*p* = 0.0001). Femoral arteries showed even more pronounced differences, with a LLL of 3.39 ± 1.22 mm in HFDN animals vs. 1.97 ± 0.43 mm in standard diet fed animals (*p* = 0.003).

**Table 1:** Results of quantitative angiography directly after intervention (post Intervention), and at 4-week follow-up (follow-up). Late lumen loss [mm] = mean lumen diameter post Intervention [mm] – minimal lumen diameter follow-up [mm]

	Standard	HFDN	*p*-value
*n coronary arteries*	15	12	
Mean lumen diameter post Intervention [mm]	3.25 ± 0.30	3.26 ± 0.25	0.92
Minimal lumen diameter follow-up [mm]	2.03 ± 0.54	1.73 ± 0.61	0.19
Late Lumen Loss [mm] (mean/min)	1.22 ± 0.45	1.53 ± 0.60	0.14
n internal iliac arteries	**10**	**8**	
Mean lumen diameter post Intervention [mm]	4.66 ± 0.24	4.66 ± 0.35	0.99
Minimal lumen diameter follow-up [mm]	3.55 ± 0.42	2.35 ± 0.70	0.0003
Late Lumen Loss [mm] (mean/min)	1.11 ± 0.47	2.32 ± 0.55	0.0001
n femoral arteries	**10**	**8**	
Mean lumen diameter post Intervention [mm]	6.27 ± 0.19	6.31 ± 0.53	0.47
Minimal lumen diameter follow-up [mm]	4.30 ± 0.46	2.92 ± 1.72	0.03
Late Lumen Loss [mm] (mean/min)	1.97 ± 0.43	3.39 ± 1.22	0.003

### Histological Analysis

**Fig. 5:**
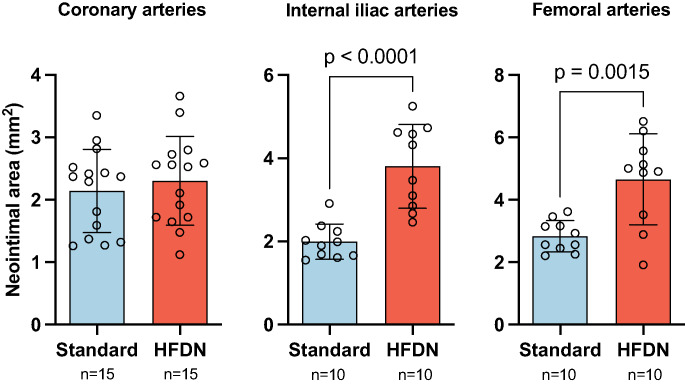
In-stent neointimal area at 4 weeks follow-up. Mean ± SD and individual data points. *N* = 15 stents per group for coronary arteries, *n* = 10 stents per group for peripheral arteries

Histological examination of peripheral arteries showed that stent implantation and vessel overstretch induced significantly increased lumen narrowing due to neointimal proliferation in HFDN compared to standard fed animals (Table 2; Fig. 5). Internal iliac arteries in HFDN animals showed significantly reduced lumen area (5.13 ± 1.56 mm^2^ vs. 7.01 ± 1.82 mm^2^; *p* = 0.02) and increased neointimal area (3.81 ± 1.01 mm^2^ vs. 2.00 ± 0.42 mm^2^; *p* < 0.0001), resulting in significantly greater lumen loss % (41.5 ± 10.3% vs. 23.4 ± 8.8%; *p* = 0.0005). Similarly, femoral arteries exhibited significantly increased neointimal area (4.66 ± 1.46 mm^2^ vs. 2.84 ± 0.50 mm^2^; *p* = 0.002) and lumen loss % (44.0 ± 16.2% vs. 24.3 ± 6.2%; *p* = 0.002) in HFDN compared to the Standard group. In coronary arteries, lumen and neointimal areas as well as lumen loss (%) were not statistically significant different between the dietary groups. Semi-quantitative vascular injury scoring demonstrated no significant differences between dietary groups across all three arterial beds (Table 2), indicating comparable procedural vessel injury despite variations in inflation pressure observed in internal iliac and femoral arteries.

**Table 2:** Results of histomorphometry and semi-quantitative injury scoring at 4-week follow-up. Lumen loss %= (neointimal area / elastica interna area)*100

	Standard	HFDN	*p*-value
*n coronary arteries*	15	15	
Lumen (mm^2^)	3.04 ± 1.17	3.15 ± 1.27	0.82
Neointima (mm^2^)	2.14 ± 0.67	2.30 ± 0.71	0.53
Lumen Loss (%)	40.5 ± 17.6	44.5 ± 18.8	0.55
Injury Score	0.48 ± 0.87	0.25 ± 0.34	0.98
n internal iliac arteries	**10**	**10**	
Lumen (mm^2^)	7.01 ± 1.82	5.13 ± 1.56	0.02
Neointima (mm^2^)	2.00 ± 0.42	3.81 ± 1.01	< 0.0001
Lumen Loss (%)	23.4 ± 8.8	41.5 ± 10.3	0.0005
Injury Score	0.02 ± 0.05	0.00 ± 0.01	> 0.9999
n femoral arteries	**10**	**10**	
Lumen (mm^2^)	8.87 ± 2.67	6.75 ± 3.04	0.11
Neointima (mm^2^)	2.84 ± 0.50	4.66 ± 1.46	0.002
Lumen Loss (%)	24.3 ± 6.2	44.0 ± 16.2	0.002
Injury Score	0.01 ± 0.19	0.07 ± 0.10	0.08

### Features of Early Neoatherosclerosis

**Fig. 6:**
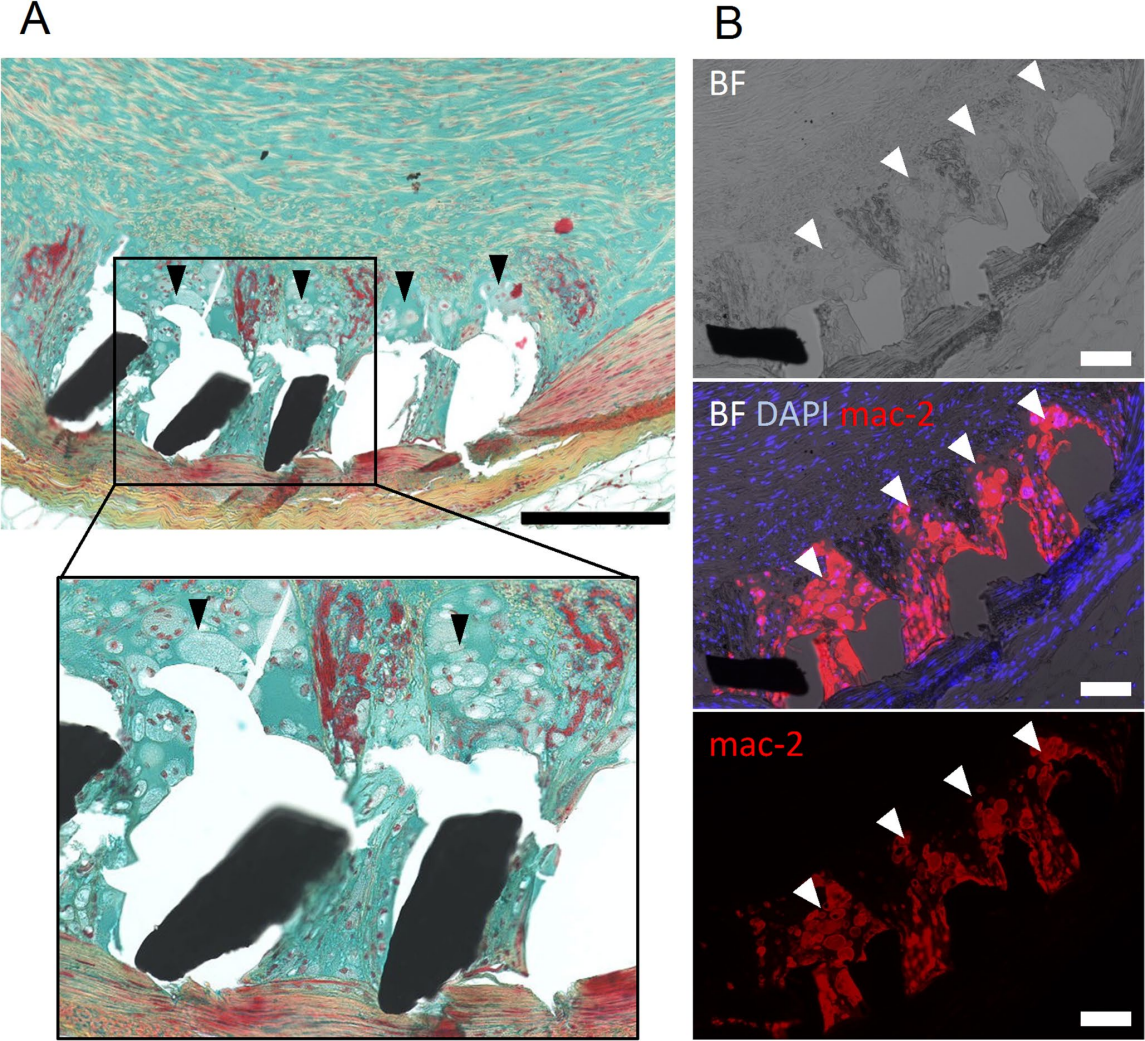
(A) Movat pentachrome stained internal iliac artery section with peri-strut foam cells (black arrows). Bar = 250 μm. (B) Mac-2 staining of a sequential section of A showing positive stained cells surrounding the stent struts (white arrows). BF = brightfield, Bar = 100 μm

Movat pentachrome-stained sections from HFDN-treated pigs revealed distinctive peri-strut accumulations of bloated, vacuolated cells with morphological characteristics consistent with foam cells (Fig. 6). Immunofluorescence staining with mac-2 antibody confirmed the presence of inflammatory macrophages within these cellular accumulations (Fig. 6). The morphological appearance and immunostaining pattern of these mac-2-positive cells differed markedly from the fluorescently labeled macrophages typically observed surrounding stent struts, as previously shown in Kamann et al. (2021) [15]. The distinctive cellular morphology and immunostaining pattern strongly suggest that these cells are foamy macrophages.

**Fig. 7:**
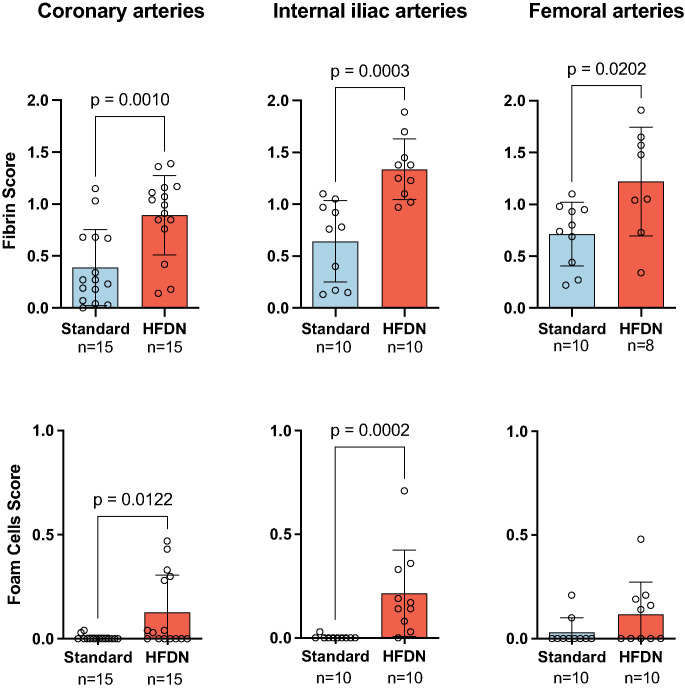
Semi-quantitative scoring of stent-strut fibrin depositions, and foam cells in standard and HFDN-fed animals. Mean ± SD and individual data points, * *p* ≤ 0.05, *** *p* ≤ 0.001. *N* = 15 stents per group for coronary arteries, *n* = 10 stents per group for peripheral arteries, except for fibrin score in A. fem. HFDN *n* = 8

Semi-quantitative scores of stent strut-associated fibrin depositions were significantly higher in arteries from HFDN-fed pigs compared to those on a standard diet across all arterial beds (Fig. 7). Peri-strut foam cell accumulations, as shown in Fig. 6A, were more pronounced in stented arterial segments of HFDN-fed pigs (Fig. 7).

**Fig. 8:**
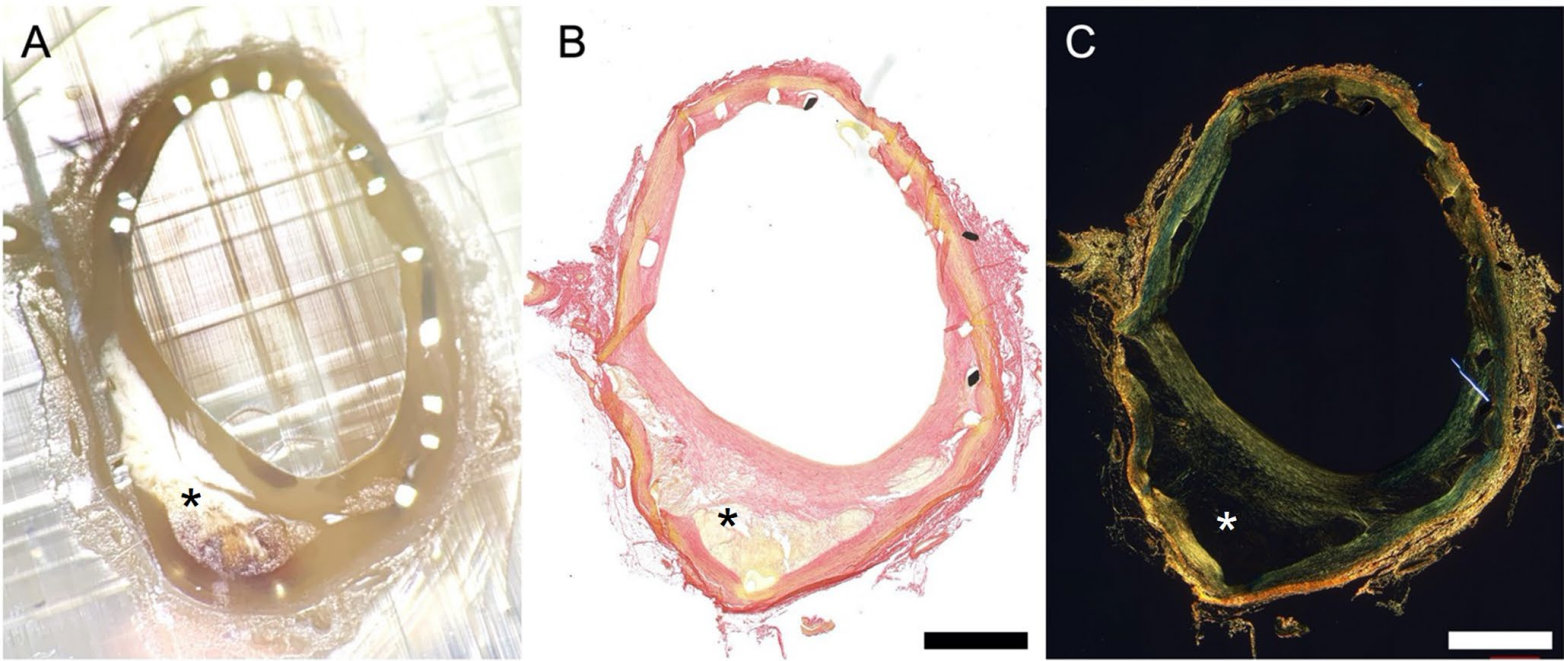
(A) Femoral artery cross-section of a HFDN-fed pig in a methyl-methacrylate block showing whitish deposition (asterisk) in the vessel wall. (B) Picro-sirius red (PSR) stained tissue section from this block and (C) PSR staining under polarized light showing a lack of collagen in the arterial wall. Bar = 1 mm

Selected stented segments of HFDN-fed pigs showed whitish depositions within the vessel wall (Fig. 8A; Table 3). Picro-sirius red staining of these sections revealed a lack of collagen within the arterial wall, a characteristic feature of early atheroma formation (Fig. 8B, C). These whitish depositions were observed exclusively in arterial segments from HFDN-fed pigs (Table 3).

**Table 3:** Segments (proximal, mid, distal) of stented arteries with whitish depositions in the vessel wall (positive segments/analyzed segments)

	Standard	HFDN
coronary arteries	0/45	6/45
internal iliac arteries	0/30	1/30
femoral arteries	0/30	4/30

### Elemental Imaging of Arterial Calcification

**Fig. 9:**
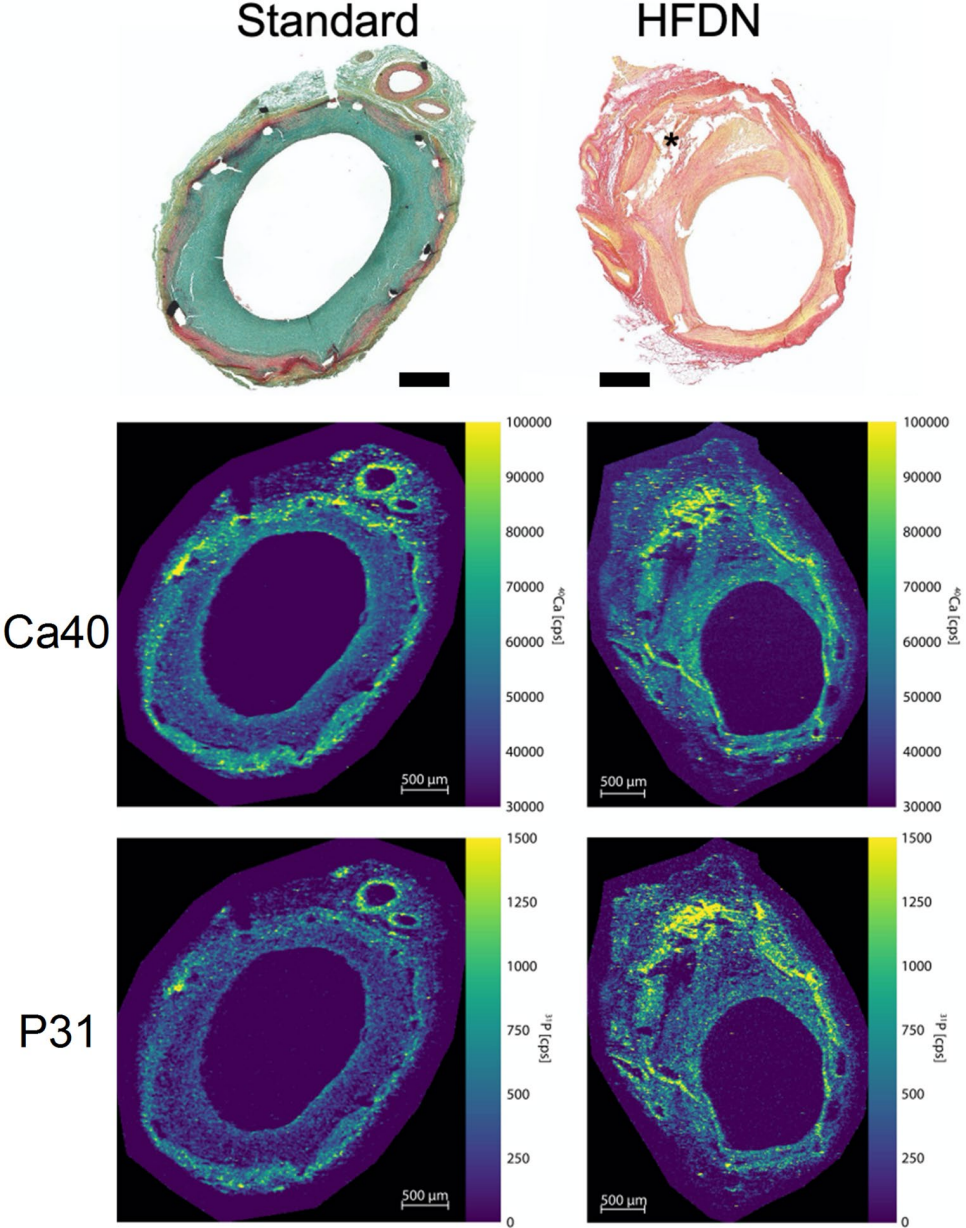
LA-ICP-MS analysis of stented LAD sections from standard-fed (left panels) and HFDN-fed (right panels) pigs. Movat pentachrome staining of a LAD section from the Standard group (upper left panel) shows concentric stenosis due to neointimal proliferation (green). PSR staining of a LAD section from the HFDN group (upper right panel) shows eccentric stenosis. Bar = 500 μm. Elemental imaging confirms increased [31] P and ^40^Ca abundance in the eccentrically thickened arterial wall (asterisk)

Elemental imaging of stented arterial sections was performed to assess calcium-phosphate depositions within the arterial wall. LA-ICP-MS scanning revealed increased levels of calcium and phosphorous in a LAD section with eccentric stenosis from the HFDN group compared to a LAD section with concentric stenosis from the standard group (Fig. 9). Notably, calcium and phosphate accumulations were primarily localized within the region of eccentric neointimal thickening.

### Ca^2+^ and Lipid Content in Unstented Arterial Segments

Arterial Ca^2+^ and lipid content was analyzed in various untreated (no stenting, no overstretch) vessel segments including aortic segments, limb arteries and internal thoracic arteries. Among all analyzed vessels, the HFDN group demonstrated a trend towards higher arterial Ca^2+^ content compared to the standard diet group (470 ± 256 mg/kg vs. 281 ± 167 mg/kg dry weight, *p* = 0.06) (Table 4). Arterial lipid content was higher in the HFDN group compared to the standard diet group (17.3 ± 11.1% vs. 13.5 ± 10.2% of dry weight, *p* = 0.41) without reaching statistical significance.

**Table 4:** Ca^2+^ and lipid content over all analyzed arterial samples. Comparison between groups was done after averaging values per animal (*n* = 5 animals per group)

	Standard	*n* arteries analyzed	HFDN	*n* arteries analyzed	t-test
Dry weight (incl. lipids) % of wet weight	24.6 ± 3.0	39	25.7 ± 4.8	31	0.17
Lipid content % of dry weight	13.5 ± 10.2	33	17.3 ± 11.1	26	0.41
Ca^2+^ content [mg/kg] of dry weight	281 ± 167	33	470 ± 256	24	0.06

## Discussion

Currently, there is no practical animal model that replicates the neoatherosclerosis-prone environment observed in patients undergoing endovascular therapy. To address this critical gap, we induced hyperlipidemia in juvenile pigs using an atherogenic diet supplemented with nicotine (HFDN), followed by stent implantation in coronary and peripheral arteries. Our findings demonstrate that this approach successfully induce a pro-neoatherosclerotic state, characterized by hyperlipidemia, accelerated in-stent stenosis in peripheral arteries, and the formation of early, lipid-rich, calcified lesions within a short timeframe.

Two weeks of HFDN feeding led to significantly elevated lipid and cotinine serum levels. Following stent implantation, this systemic environment translated into a distinct pathological response. At four weeks follow-up, the HFDN group showed significantly greater late lumen loss (LLL) and neointimal proliferation in peripheral arteries compared to pigs on a standard diet. Histological analysis confirmed these findings and, more importantly, revealed hallmark features of early neoatherosclerosis like peri-strut foam cell accumulations, increased fibrin deposition, and the development of collagen-deficient, calcified lesions. The detection of increased calcium-phosphate in these lesions via elemental imaging indicates the initiation of pathological arterial mineralization. Additionally, untreated (native) arterial segments in the HFDN group exhibited a higher calcium content (470 ± 256 vs. 281 ± 167 mg/kg dry weight, *p* = 0.06), suggesting a pro-atherogenic environment that contributes to disease progression. Although arterial calcium and lipid contents in unstented vessels did not reach statistical significance, both parameters showed a consistent numerical increase in the HFDN group. Given the limited sample size and the relatively short induction period (6 weeks of HFDN feeding in total), these findings may reflect early changes that are not yet fully developed; extended feeding or longer follow-up may further amplify these differences, but this was beyond the scope of the present pilot study.

Balloon inflation pressures were individually adjusted to achieve a standardized vessel overstretch of approximately 20%, not predefined per group. Therefore, minor differences in mean inflation pressures in peripheral arteries (Standard vs. HFDN: internal iliac 11.1 ± 1.0 vs. 9.8 ± 1.4 atm, femoral 12.0 ± 0.0 vs. 10.8 ± 1.0 atm; Supplementary Table S2) reflect inter-individual variations in vessel size and compliance rather than systematic procedural differences between groups. These modest pressure differences are unlikely to contributed to the observed restenotic or neoatherosclerotic responses, as evidenced by comparable injury scores (Table 2) between groups.

### Nicotine Supplementation

Early research demonstrated that pigs tolerate oral nicotine supplementation up to approximately 4 mg/kg body weight/day [19]. Nicotine combined with vitamin D overload induced vascular calcifications in rats [20]. In more recent studies, intramuscular nicotine injections accelerated atherosclerosis in balloon-injured porcine coronaries [21]. Incorporating nicotine (1 mg/kg body weight/day) directly into the atherogenic diet, offers an advantage over multiple muscular injections by reducing workload, as well as minimizing animal stress and pain. The administered dose was well-tolerated, with no impact on animal behavior and blood pressure. Lower weight gain compared to animals fed with piglet-raising diet might be due to the known appetite-suppressing effects of nicotine but had no impact on animal well-being. Blood and urine cotinine measurements confirmed the systemic nicotine exposure and metabolization.

### Vascular Responses

The HFDN regimen, which elevated serum LDL and cotinine levels, significantly increased in-stent late lumen loss in internal iliac (2.32 ± 0.55 vs. 1.11 ± 0.47 mm, *p* = 0.0001) and femoral arteries (3.39 ± 1.22 vs. 1.97 ± 0.43 mm, *p* = 0.003) compared to the Standard group, while coronary arteries showed no significant differences in late lumen loss (1.53 ± 0.60 vs. 1.22 ± 0.45 mm, *p* = 0.014) or neointima (Tables 1 and 2; Fig. 5). Numerous studies indicate that hemodynamic, structural, and cellular differences between vascular beds contribute to differential pathological responses, although the precise mechanisms remain unclear [22]. While mechanistic data are lacking in the present study, differences in vessel wall structure, smooth muscle cell content, and biomechanical stress exposure in peripheral arteries may contribute to an enhanced remodeling response [23]. Repetitive deformation associated with limb movement could exacerbate endothelial dysfunction and inflammatory cell recruitment in the pro-atherogenic HFDN setting, thereby promoting smooth muscle proliferation and stenosis within peripheral stents [24]. Epidemiologic data show smoking exerts a disproportionately greater risk for peripheral arteries disease (PAD) than coronary artery disease. Accordingly, the HFDN-induced hyperlipidemia and nicotine exposure, combined with the unique biomechanical and hemodynamic environment of peripheral vessels likely recapitulate this heightened PAD susceptibility observed clinically [25, 26].

### Positioning Within the Preclinical Model Landscape

The current landscape of preclinical models for NA is diverse and expanding [9, 27], ranging from genetically modified mice to rabbits and pigs. Each model offers a unique balance of clinical relevance and practicality. Our HFDN porcine model contributes to this landscape by offering a practical, large-animal platform. Its principal advantages are the use of human-sized endovascular devices and the ability to assess vascular responses in both coronary and peripheral beds, which are known to differ in their reaction to injury and treatment in preclinical models [15, 23] and in disease manifestation in patients [28, 29]. Simultaneous evaluation across multiple vascular beds under hyperlipidemic and nicotine exposure allows for the investigation of vascular bed–specific responses beyond traditional restenosis models, moving toward the translational setting of revascularization in an atherosclerotic milieu.

### Limitations

This pilot study has several limitations. First, the number of animals per group was modest, and one HFDN animal died before 4-week follow-up, which limits statistical power for some endpoints and may have contributed to borderline findings such as arterial calcium content in unstented vessels. Second, the 4-week follow-up was designed to capture early neoatherosclerotic changes. More advanced lesions with necrotic cores were infrequent, which potentially restricts conclusions regarding devices targeting late-stage neoatherosclerosis. Third, molecular characterization was limited to histomorphology, mac-2 immunostaining, and elemental imaging. Dedicated staining for mechanistic markers was not performed. This first study only incorporated uncoated devices (uncoated balloons in bare metal stents), following studies will implement drug coated devices.

### Clinical Perspective

Neoatherosclerosis has emerged as a major determinant of late stent failure and adverse clinical events in both coronary and peripheral interventions, particularly in patients with persistent risk factor exposure such as hyperlipidemia and smoking [30–34]. The HFDN porcine model reproduces a clinically relevant combination of elevated LDL levels, systemic nicotine exposure, and early neoatherosclerotic lesion formation within a short experimental timeframe. Accordingly, this model provides a practical and translational platform for evaluating drug-coated and bioresorbable devices, supporting the development of novel revascularization strategies aimed at improving long-term patient outcomes.

## Conclusion

By combing a high-fat, nicotine-supplemented diet (HFDN) and bare mental stent deployment in juvenile domestic pigs, the current protocol successfully stimulates a hyperlipidemic, pro-inflammatory environment that fosters the development of early neoatherosclerotic features including macrophages infiltration, peri-struct foam cell accumulation, increased fibrin deposition, collagen-poor lesions and pathological microcalcification within a 4-week timeframe. This HFDN porcine model combines practical feasibility with the use of human-sized devices and reproduces clinically relevant vascular bed-specific responses, providing a versatile platform for the preclinical evaluation of vascular therapies.

## Supplementary Information

Below is the link to the electronic supplementary material.

Supplementary Material 1

Supplementary Material 2

## Data Availability

The datasets generated and/or analyzed during the current study are available from the corresponding author on request.
