# Supplementary material for: Towards Neoatherosclerosis: A Porcine Model for Enhanced Device Testing
Source: J Cardiovasc Transl Res. 2026 May 26;19(1):56. doi: 10.1007/s12265-026-10778-4 (PMC13212638; doi:10.1007/s12265-026-10778-4)
Supplement: Supplementary file 2 — Supplementary Material 2 [file 12265_2026_10778_MOESM2_ESM.doc]

Supplement

Supplemental Table S1: Composition of diets

Group	Standard diet	HFD + Nicotine
Diet	deuka primo plus	Altromin 900015+nicotine
Fat (%)	4.2	20
Cholesterol (%)	-	2.0
Sodium-Cholate (%)	-	0.5
Calcium (%)	0.75	4.0
Phosphate (%)	0.55	3.0
Vitamin D3 (IE/kg)	2000	4000
L-Methionine (%)	0.4	2.3
Nicotine (ppm)	-	30

Supplemental Table S2: Number of balloons, sizes and inflation pressures used for stent deployment at initial intervention. Pressures were individually adjusted to achieve ~20% vessel overstretch based on angiographic vessel diameter and balloon compliance charts.

	Coronary arteries		*Internal iliac arteries*		*Femoral arteries*	
	n: Balloon diameter (mm)	Inflation pressure (atm)	n: Balloon diameter (mm)	Inflation pressure (atm)	n: Balloon diameter (mm)	Inflation pressure (atm)
Standard	15 arteries: 3.5-20 3.0-20	10.1±2.3	10 arteries: 5.0-19	11.1±1.0 *	10 arteries: 6.0-18	12.0±0.0*
HFDN	15 arteries: 3.5-20 3.0-20	10.7±2.0	10 arteries: 5.0-19	9.8±1.4	10 arteries: 6.0-18	10.8±1.0

* p<0.05 vs. HFDN

Supplemental Table S3: Systolic/diastolic blood pressures directly before stent implantation and at 4-week follow-up (FU)

Group	Animal	before stent implantation (mmHg)	4-week FU (mmHg)	BP change
Standard	1	93/66	85/63	+8/-3
	2	127/104	104/82	-23/-12
	3	109/82	119/94	+10/+12
	4	88/64	90/66	+2/+2
	5	97/70	118/84	+21/+14
HFDN	6	103/67	-	-
	7	102/63	110/58	+8/-5
	8	113/72	109/64	-4/-8
	9	81/54	103/73	+22/+19
	10	103/74	89/52	-14/-12

Supplemental Table S4: Serum lipids, blood counts and cotinine levels at stent implantation (after 2 weeks of feeding HFDN or standard diet) and 4-week follow-up (after 6 weeks of feeding HFDN or standard diet). Blood parameters were analyzed using a mixed-effects model with factors "time" and "treatment," followed by Sidak's multiple comparison test. Definitions: n.d. = not detected, n.m. = not measured, n.a. = not applicable.

		**Standard diet**						**HFDN diet**						**HFDN dietMixed-effects analysis**	
		Implantation			4-week FU			Implantation			4-week FU			$p < 0.05$	
		MW		SD	MW		SD	MW		SD	MW		SD	Time	Treatment
HD-Chol.	mmol/l	0.91	±	0.12	0.84	±	0.15	2.37	±	0.18	2.08	±	0.52	no	Treatment
**LDL-Chol**	mmol/l	1.08	±	0.13	0.98	±	0.10	3.76	±	0.57	2.73	±	1.47	no	yes
LDL-CholLDL/HDL		1.19	±	0.16	1.19	±	0.21	1.60	±	0.30	1.24	±	0.43	no	yes
**Cholesterol**	mmol/l	1.88	±	0.22	1.82	±	0.24	6.36	±	1.98	5.10	±	1.15	no	no
Free Fatty Acids	mmol/l	0.41	±	0.13	0.38	±	0.09	0.33	±	0.09	0.74	±	0.18	yes	yes
**Triglycerides**	mmol/l	0.75	±	0.18	0.65	±	0.21	0.59	±	0.37	1.07	±	0.10	no	yes
Erythrocytes	T/l	6.31	±	0.49	6.16	±	0.63	6.09	±	0.38	4.99	±	0.37	no	no
**Leukocytes**	G/l	16.42	±	4.08	15.36	±	4.61	19.66	±	4.48	16.78	±	2.99	no	yes
Thrombocytes	G/l	398.40	±	110.81	305.00	±	161.59	373.20	±	87.66	550.50	±	39.57	no	no
**Hematocrit**	l/l	0.33	±	0.02	0.31	±	0.02	0.34	±	0.02	0.29	±	0.03	yes	no
**Hemoglobin**	g/l	109.80	±	7.40	102.80	±	7.66	103.00	±	6.96	89.75	±	6.45	yes	no
**Cotinine**	ng/ml		n.d.			n.d.		28.47	±	12.91	36.21	±	5.86	n.a.	
Cotinine urine	ng/ml		n.m.		31.36	±	16.55		n.m.		3612.37	±	531.66	n.a.	

Supplemental Table S5: Instrument settings and data acquisition conditions for LA-ICP-ToF-MS imaging

NWRimage Laser Ablation system settings	
Fluence	10 J cm^{-2}
Repetition rate	100 Hz [a]
Beam size	10 μm
Mask shape	Square
Scan velocity	1000 μm s^{-1} [a]
He chamber gas flow	0.30 L min^{-1}
He cup gas flow	0.30 L min^{-1}
Wash-out time (FW0.01M)	10 ms
icpToF 2R settings	
RF power	1550 W
Ar cool flow	14 L min^{-1}
Ar auxiliary flow	0.8 L min^{-1}
Ar nebulizer flow	0.95 L min^{-1}
Sampling depth	4 mm
CRC H_2/He gas flow	4 mL min^{-1}

Supplemental Method M1: Procedures at stent implantation and 4-week follow-up

Dual antiplatelet therapy (Clopidogrel, Acetylsalicylic acid), prevention of vascular spasm during procedure (long-acting Verapamil), sedation (ketamine, xylazine hydrochloride), general anesthesia (propofol, isoflurane), pain medication, and vascular access via the common carotid artery are described elsewhere [13]. Ursocyclin (20 mg/kg, i.m.) was administered as antibiotic therapy. Daily acetylic acid (100 mg/kg) and Clopidogrel (75 mg/kg) were given orally until end of study.

Imaging was performed using a Siemens AXIOM Artis zee fluoroscope. Under fluoroscopic control a guiding catheter was introduced through the arterial sheath over a guidewire into the aorta. Arteries were visualized using an approved nonionic iodinated contrast agent. For purpose of QA radiographs of native vessels, images with stents, and inflated balloons were taken as well as results after treatment of each artery, and at 4-week follow-up. Throughout the procedure, electrocardiogram (ECG), SpO_2 and body temperature were monitored continuously. At 4-week follow up, the animals were treated and anesthetized as described above, except for the antibiotic therapy. Angiography was performed for offline evaluation of stent deformation and quantitative angiography (QA). After final angiography the animals were euthanized in deep anesthesia using an intravenous bolus of 10 ml super-saturated potassium chloride. Directly afterwards samples for analyses were taken. All tissue samples for histology

Supplemental Method M1: Procedures at stent implantation and 4-week follow-up

Dual antiplatelet therapy (Clopidogrel, Acetylsalicylic acid), prevention of vascular spasm during procedure (long-acting Verapamil), sedation (ketamine, xylazine hydrochloride), general anesthesia (propofol, isoflurane), pain medication, and vascular access via the common carotid artery are described elsewhere [13]. Ursocyclin (20 mg/kg, i.m.) was administered as antibiotic therapy. Daily acetylic acid (100 mg/kg) and Clopidogrel (75 mg/kg) were given orally until end of study.

Imaging was performed using a Siemens AXIOM Artis zee fluoroscope. Under fluoroscopic control a guiding catheter was introduced through the arterial sheath over a guidewire into the aorta. Arteries were visualized using an approved nonionic iodinated contrast agent. For purpose of QA radiographs of native vessels, images with stents, and inflated balloons were taken as well as results after treatment of each artery, and at 4-week follow-up. Throughout the procedure, electrocardiogram (ECG), SpO_2 and body temperature were monitored continuously. At 4-week follow up, the animals were treated and anesthetized as described above, except for the antibiotic therapy. Angiography was performed for offline evaluation of stent deformation and quantitative angiography (QA). After final angiography the animals were euthanized in deep anesthesia using an intravenous bolus of 10 ml super-saturated potassium chloride. Directly afterwards samples for analyses were taken. All tissue samples for histologywere rapidly fixed with 10% buffered formalin. Tissue samples for lipid and Ca^{2+} analyses were frozen at -20°C until usage.
