# Supplementary material for: Towards Neoatherosclerosis: A Porcine Model for Enhanced Device Testing
Source: J Cardiovasc Transl Res. 2026 May 26;19(1):56. doi: 10.1007/s12265-026-10778-4 (PMC13212638; doi:10.1007/s12265-026-10778-4)
Supplement: Supplementary file 1 — Supplementary Material 1 [file 12265_2026_10778_MOESM1_ESM.doc]

September 8th, 2025

Dear Editor:

We are pleased to submit our manuscript entitled “Towards Neoatherosclerosis: A Porcine Model for Enhanced Device Testing” for consideration in the *Journal of Cardiovascular Translational Research*.

This study addresses a critical gap in preclinical cardiovascular research by establishing a reproducible large animal model that simulates the hyperlipidemic, pro-atherogenic conditions seen in patients undergoing endovascular interventions. Using a short protocol in commercially available juvenile pigs, we combined a high-fat, nicotine-supplemented diet with coronary and peripheral stent implantation. The model reliably induces hyperlipidemia, in-stent stenosis, foam cell accumulation, and intimal calcium deposition, which are hallmarks of early neoatherosclerosis. This accessible and time-efficient approach provides a clinically relevant platform for evaluating endovascular devices without requiring prolonged induction periods or genetically modified animals. This is particularly relevant as growing clinical awareness highlights that drug-coated devices may inadvertently promote neoatherosclerosis and late stent failure, underscoring the urgent need for preclinical platforms that allow thorough evaluation of device-associated neoatherosclerosis formation. That’s why our findings will be of broad interest to researchers and clinicians in the fields of cardiovascular device development, interventional cardiology/radiology, and translational atherosclerosis research.

This work has not been published or submitted elsewhere. The authors have no conflicts of interest to declare that are relevant to the content of this article. Please address all correspondence concerning our manuscript to [tobias.haase@charite.de](mailto:tobias.haase@charite.de).

Yours sincerely,

Tobias Haase
